# The Significance of MicroRNAs Expression in Regulation of Extracellular Matrix and Other Drug Resistant Genes in Drug Resistant Ovarian Cancer Cell Lines

**DOI:** 10.3390/ijms21072619

**Published:** 2020-04-09

**Authors:** Dominika Kazmierczak, Karol Jopek, Karolina Sterzynska, Barbara Ginter-Matuszewska, Michal Nowicki, Marcin Rucinski, Radoslaw Januchowski

**Affiliations:** Department of Histology and Embryology, Poznan University of Medical Sciences, PL-61-781 Poznan, Poland; dominika.ka.poznan@gmail.com (D.K.); karoljopek@ump.edu.pl (K.J.); k.olejniczak@ump.edu.pl (K.S.); bginter@man.poznan.pl (B.G.-M.); mnowicki@ump.edu.pl (M.N.); marcinruc@ump.edu.pl (M.R.)

**Keywords:** microRNA, ATP-binding cassette subfamily B member 1 gene (*ABCB1*), collagen type III alpha 1 chain gene (*COL3A1*), ovarian cancer, drug resistance

## Abstract

Ovarian cancer rates the highest mortality among all gynecological malignancies. The main reason for high mortality is the development of drug resistance. It can be related to increased expression of drug transporters and increased expression of extracellular matrix (ECM) proteins. Our foremost aim was to exhibit alterations in the miRNA expression levels in cisplatin (CIS), paclitaxel (PAC), doxorubicin (DOX), and topotecan (TOP)—resistant variants of the W1 sensitive ovarian cancer cell line—using miRNA microarray. The second goal was to identify miRNAs responsible for the regulation of drug-resistant genes. According to our observation, alterations in the expression of 40 miRNAs were present. We could observe that, in at least one drug-resistant cell line, the expression of 21 miRNAs was upregulated and that of 19 miRNAs was downregulated. We identified target genes for 22 miRNAs. Target analysis showed that miRNA regulates key genes responsible for drug resistance. Among others, we observed regulation of the ATP-binding cassette subfamily B member 1 gene (*ABCB1*) in the paclitaxel-resistant cell line by miR-363 and regulation of the collagen type III alpha 1 chain gene (*COL3A1*) in the topotekan-resistant cell line by miR-29a.

## 1. Introduction

Ovarian cancer poses a growing threat to women’s lives and health and occupies the first place in mortality among gynecological cancers [1]. Most cases of ovarian cancer are diagnosed at a very advanced stage (III or IV according to the International Federation of Gynaecology and Obstetrics (FIGO)) [2,3]. The molecular basis of ovarian cancer is very complicated, as ovarian cancer is very heterogeneous with different histological types. Even in the same histological type, a different profile of cancer-related genes among individual cases can be observed [4]. Despite the dynamic development of gynecologic oncology, the main method of ovarian cancer treatment is the surgical treatment followed by chemotherapy [5]. The first line treatment is based on combination platinum derivatives (carboplatin or cisplatin) and taxanes (paclitaxel—PAC) [2]. The basis for choosing the second line chemotherapy regimen is sensitivity to treatment with platinum derivatives, which determines the prognosis [2]. Low chemotherapy effectiveness result from primary or developed during treatment drug resistance [6]. In over 80% of patients, it comes to recurrence cancer [2]. There are two main types of mechanisms of drug resistance: cellular specific and tissue specific [7]. A cellular mechanism is based on cytostatics active removal from cancer cells. Proteins from the ATP-binding cassette (ABC) family contribute to this phenomenon [8]. Cellular mechanisms also include repairing damaged DNA, developing point mutations in the genes that encode proteins that bind cytostatic drugs, and increasing the activity of anti-apoptotic or pro-survival pathways as well as disrupting apoptotic signaling pathways [5]. On the other hand, the tissue specific mechanisms are associated with tumor vascularization, cell density in the tumor, and expression of extracellular matrix proteins (ECM) [9]. All of these change drug distribution in the tumor tissue, decreasing their availability to tumor cells. Additionally, ECM proteins can induce cell adhesion mediated drug resistance (CAM-DR), leading to increased resistance to apoptosis [10]. Although many genes associated with the development of resistance to chemotherapy are known, the mechanisms of their regulation are still poorly understood. One of the ways to regulate gene expression is regulation at the mRNA level by small noncoding RNA particles, designated as a micro RNA (miRNA). Short, non-coding RNAs were discovered and described for the first time in 1993 by Victor Ambros from Harvard University [11]. miRNAs are short (19–29 nucleotides), single-stranded, non-coding RNA molecules with a phosphate residue (5′ end) and a hydroxyl group (3′ end) [12]. They play an important regulatory role in the expression of genes in both animals and plants [12]. In the case of miRNA, the regulation of gene expression takes place at the post-transcriptional level and involves interaction with mRNA [13]. The seed sequence (6–8 nucleotides) located in the 5′ region of the miRNA plays a key role in this process. miRNA seed recognizes the sequence of the target mRNA complementary within the 3′ UTR (untranslated region). Depending on the degree of complementarity of the mRNA to the mature miRNA, either the transcript degradation (complete or almost complete complementarity) occurs or the translation is inhibited (incomplete complementarity) [12]. The effect of miRNA expression can be pleiotropic. Modulation of one miRNA expression can affect many different genes responsible for the various mechanisms of cancer [14]. The sequences encoding miRNAs in the human genome can be located both in introns of genes and in non-endogenous areas. Some of the miRNA encoding genes may also be located within exons of other genes. miRNAs are classified in groups called miRNA families, and most of them are transcribed by RNA polymerase II [15]. Family members are characterized by common origin, evolutionary conservation in sequence, similar mature miRNA/miRNA* (shared functional characteristics or biological function), and conserved mature miRNA–seed–target relationships [16,17]. It is very interesting that miRNA genes in the same miRNA family are non-randomly localized around genes connected with cancer, infectious, immune system, sensory system, neurodegenerative diseases, and development [16]. A set of two or more [18] miRNAs transcribed from physically adjacent miRNA genes is called a cluster of miRNA. miRNAs that create the same cluster are transcribed in the same orientation without separation by any transcription unit. Clusters of miRNAs take part in controlling various important cellular processes such as tumor formation and development of lungs, heart, and immune system [18]. Changes in miRNA expression have been reported in many diseases, including different cancers. An miRNA profiling study showed that the pattern of miRNA expression in cancer tissues compared to normal tissues was very different [19]. miRNA can perform suppressor or oncogenic functions in cancers. As a tumor suppressor, it may inhibit cancer growth by negative oncogenes regulation; generally, suppressor miRNA expression is decreased in cancer cells [20]. On the contrary, the oncogene miRNAs expression (called “oncomirs”) is increased in tumors. Oncomirs promote tumors development and progression by the mechanism of downregulating a tumor suppressor or other genes involved in, for example, cell proliferation or cell differentiation in cancer [19]. In ovarian cancer, expression of different suppressors (miR-26b [21], miR-29b [22], miR-30a-5p [23], miR-93-5p [24], miR-106b-5p [25], miR-125b [26]) and oncogenes (miR-183 [27], miR-376a [28], miR-383-5p [29], miR-551b-3p [30], miR-572 [31]) was described. Ovarian cancer tissue studies revealed the negative correlation between members of the miR-23 family. High expression level of miR-23a and low expression level of miR-23b were connected with the stage and the degree of cancer, the differentiation level, and the lymph nodes metastases [32]. Much attention is currently being paid to miRNA in the context of drug resistance in ovarian cancer [13,19,20]. Changes in miRNA expression are associated with the development of resistance to chemotherapy in patients [33] as well as in vitro in the sensitive-resistant cell line model [33]. In PAC-resistant patients, downregulation of miR-663 and miR-622 was correlated with better prognosis [33]. Overexpression of miR-647 in the PAC-sensitive patients was correlated with good prognosis, which suggested suppressor function of miR-647 [33]. Changes in miRNA expression were also observed in the cell line study. Downregulation of miR-31 expression correlated with taxane resistance in ovarian cancer cell lines [34]. In contrast, upregulation of miR-98-5p was observed in cisplatin (CIS)-resistant cell lines [35]. Changes in miRNAs expression were also noted in another cancer. miR-195 expression was downregulated in temozolomid-resistant glioma cells [36], and miR-203 was downregulated in prostate cancer cells resistant to doxorubicin (DOX) [37]. The use of miRNA microarrays to analyze changes in miRNAs expression is an effective molecular tool for discovering new miRNAs involved in drug resistance processes. The present study shows alterations in the miRNA expression levels in CIS (W1CR), PAC (W1PR1 and W1PR2), DOX (W1DR), and topotecan (TOP) (W1TR) resistant variants of the W1 sensitive ovarian cancer cell line. We also identified a set of target genes that can be responsible for drug resistance in these cell lines.

## 2. Results

### 2.1. Gene Chip Quality Assessment

In the present study, we used standard factors such as signal-to-noise ratio internal hybridization and controlled spike-in-controls to preliminarily determine the quality of analyzed samples. Controlled spike-in-controls were: spike_in-control-2, spike_in-control-23, spike_in-control-29, spike_in-control-31, spike_in-control-36. Oligos 2, 23, and 29 are RNA, confirming the poly(A) tailing and the ligation. Oligo 31 (poly(A) RNA) confirmed ligation. Oligo 36 is poly(day) DNA and confirmed ligation and the lack of RNases in the RNA sample.

### 2.2. Gene Expression Evaluation and Gene Expression Lists

We evaluated changes in the transcription level of miRNAs. Analysis of miRNA expression in five drug resistant ovarian cancer cell lines provided new information about the significance of changes in miRNAs expression in drug resistance development in ovarian cancer. Table 1 summarizes the changes in the expression of miRNAs in drug resistant sublines with respect to the drug sensitive W1 cell line. Statistically significant level changes higher than 5-fold and less than 0.2-fold (up-/down-regulation of more than/less than 5 and −5, respectively) in the drug-resistant cells relative to those in their drug-sensitive counterparts were evaluated. miRNAs with expression levels of between 5- and 0.2-fold of those of the controls were considered “not significant (NS)” when the gene lists were constructed.

### 2.3. miRNAs Expression in Drug Resistant Cell Lines

**Figure 1:**
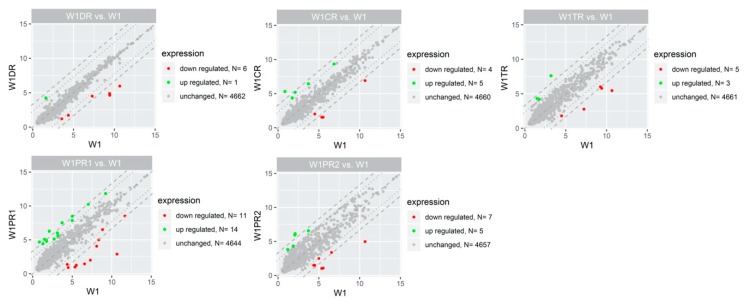
Scatter plots displaying the miRNA with expression levels that were upregulated (green dots) or downregulated (red dots) by 5-fold or more in drug resistant cells in relation to the W1 cells. Grey dots indicate miRNAs below cut-off criteria (|fold|> 5, *p* < 0.05).

The general profile of miRNA expression in the appropriate experimental group is presented in Figure 1.

**Figure 2:**
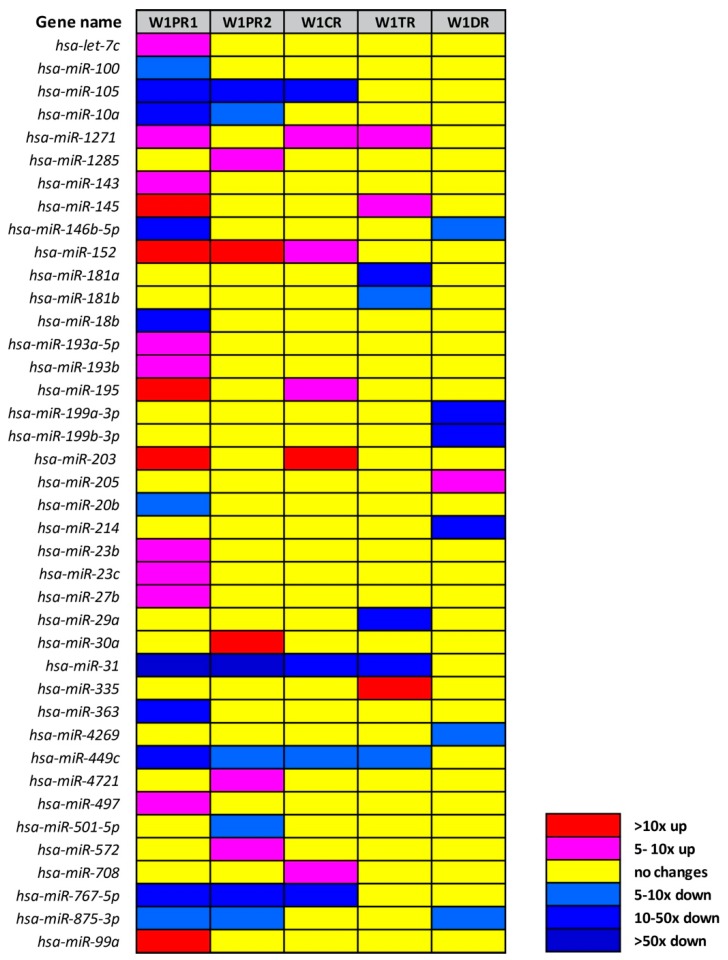
Expression ratios of miRNAs in drug-resistant sublines.

We observed alterations in the expression of 40 miRNAs (Table 1, Figure 2). The level of expression of 21 miRNAs was upregulated in at least one drug resistant cell line. Expression of 19 miRNAs was downregulated in at least one drug resistant cell line.

In relation to the determined cut-off criteria (fold ±5, *p* < 0.05), the most alternations were observed for W1PR1 (PAC resistant cell line), which showed 25 miRNAs with altered expression (14 miRNAs upregulated and 11 miRNAs downregulated). Changes in expression of 12 miRNAs were observed for W1PR2 cell line with five genes upregulated and seven genes downregulated. Much fewer changes were observed for other drug resistant cell lines. In the CIS resistant cell line, we observed changes in nine miRNAs (five miRNAs upregulated and four miRNAs downregulated). In the TOP resistant cell line, changes in eight miRNAs expressions were observed (three miRNAs upregulated and five miRNAs downregulated), and finally, in the DOX-resistant cell line, we observed changes in the expression of seven miRNAs (one miRNA upregulated and six miRNAs downregulated).

Based on the following standards, we selected 27 of 40 miRNAs for further analysis: (1) miRNAs with changes in expression at least 5-fold (up or down) in at least three drug resistant cell lines; (2) at least 10-fold changes in miRNAs expression in one drug resistant cell line; (3) 5-fold changes in expression of at least two miRNAs from the same family. Expression of miR-31 and miR-449c changed in four drug resistant cell lines (W1PR1, W1PR2, W1CR, and W1TR). Expression of miR-105, miR-1271, miR-152, miR-767-5p, and miR-875-3p changed in three drug resistant cell lines. Expression of 12 miRNAs was changed at least 10-fold in one tested line, and between them, expression of miR-10a, miR-146b-5p, miR-18b, miR-214, miR-29a, and miR-363 was downregulated, and expression of miR-145, miR-195, miR-203, miR-30a, miR-335, and miR-99a was upregulated.

Expression of miRNA from families 181 (miRNA-181a and miRNA-181b), 193 (miRNA-193a-5p and miRNA-193b), 199 (miRNA-199a-3p and miRNA-199b-3p), and 23 (miRNA-23b and miRNA-23c was also changed in investigated cell lines. Importantly, the expression of miRNAs belonging to the same family was altered in the same way. In particular, members of 193 and 23 families were upregulated in the W1PR1 cell line and members of 181 and 199 families were downregulated in the W1TR and the W1DR cell lines, respectively.

A high similarity between cell lines resistant to PAC was observed. In both cell lines, the same six miRNAs (miR-105, miR-10a, miR-31, miR-449c, miR-767-5p, and miR-875-3p) were downregulated, and miR-152 was upregulated. Some similarity was also observed between both PAC and CIS resistant cell lines—the drug used in the first line chemotherapy of ovarian cancer.

Of the 27 miRNAs selected for analysis, nine showed a very significant level of expression change with >20-fold. miR-31 was downregulated in four cell lines (W1PR1, W1PR2, W1CR, and W1TR) with very strong downregulation of more than 217-fold in the W1PR1 cell line and strong downregulation of 52-fold in the W1PR2 cell line and 36-fold in the W1TR cell line. Among other significantly downregulated miRNAs, we observed downregulation of miRNA-10a (34-fold), miRNA-146b-5p (39-fold), and miRNA-767-5p (20-fold) in the W1PR1 cell line. We also observed a significant downregulation of miR-105 in the W1PR2 (22-fold) cell line and miRNA-29a (22-fold) in the W1TR cell line as well as downregulation of miRNA-199a (23-fold), 199b (28-fold), and miRNA-214 (26-fold) in the W1DR cell line. Significant upregulation of miRNA-203 (22-fold) was observed in the W1CR cell line and of miRNA-335 (21-fold) in the W1TR cell line.

### 2.4. Analysis of Target Genes Expression

In the second part of our investigation, we were interested in whether described miRNAs are involved in the regulation of genes responsible for drug resistance development. By the assumption that increasing miRNA expression leads to decreasing target gene expression and vice versa, for further analysis, we selected those target genes in which the fold change was inversely correlated with the fold change of miRNA and changes at least five times up/down, respectively, with adj.p.val. <0.05. Targets with expression levels between 5- and 0.2-fold (up/down regulation between 5 and −5) compared to those of the controls were considered “not significant (NS)” when the target gene lists were constructed. For analysis of target genes expression, we used our microarray data that were published previously [38,39,40]. From target genes analysis, we excluded miRNAs expressed only in the W1PR2 cell line, because we do not possess mRNA microarray data for this cell line. Therefore, the following miRNAs were excluded from analysis: miR-1285, miR-30a, miR-4721, miR-501-5p, and miR-572. Using our criteria (changes in expression 5-fold up or down), we did not find any targets for the following miRNA: miR-1271, miR-152, miR-203, miR-335, and miR-214.

For further analysis, we selected genes involved in drug resistance, extracellular matrix, and cancer stem cell biology using the following key words in the Gene Ontology (GO) Database: response to drug, drug transport, extracellular space, extracellular matrix, collagen containing extracellular matrix, stem cell; previously, we described changes in expression of genes from these groups in investigated drug resistance cell lines [37,38,39] Using these criteria, we did not find targets for the following miRNA: miR-100, miR-10a, miR-143, miR-193a-5p, miR-199a-3p, miR-199b-3p, miR-4269, and miR-99a.

**Figure 3:**
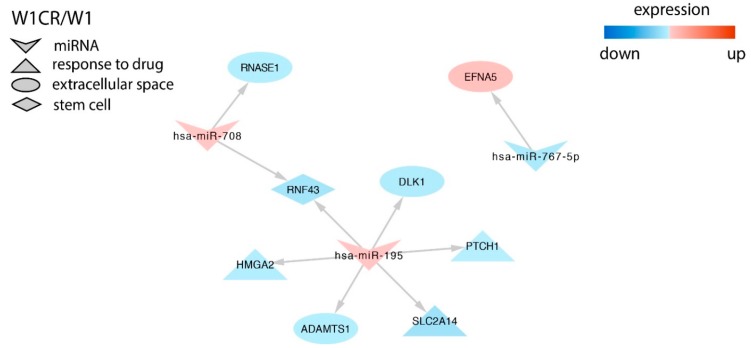
Regulation of selected target genes by miRNAs in the W1CR cell line.

In the CIS resistant cell line, we identified targets for miR-767-5p, miR-195, and miR-708. Among them, miR-195 overexpression correlated with *SLC2A14* (solute carrier) transporter downregulation (Figure 3).

**Figure 4:**
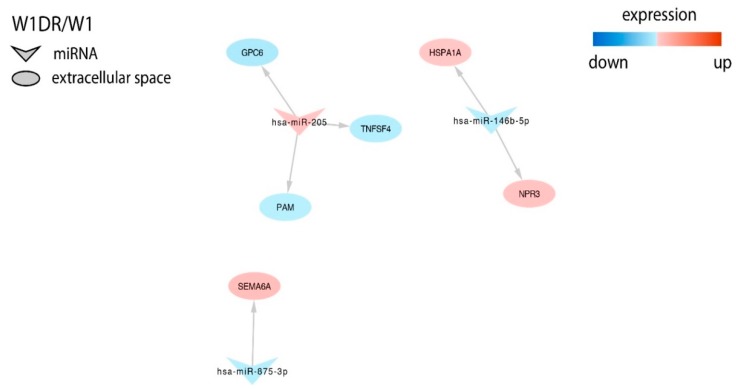
Regulation of selected target genes expression by miRNAs in the W1DR cell line.

In the DOX resistant cell line, we found targets for miR-146b-5p, miR-205, and miR-875-3p, and, among them, we could distinguish *Semaphorin 6A* (*SEMA6A*) regulated by miR-875-3p (Figure 4).

**Figure 5:**
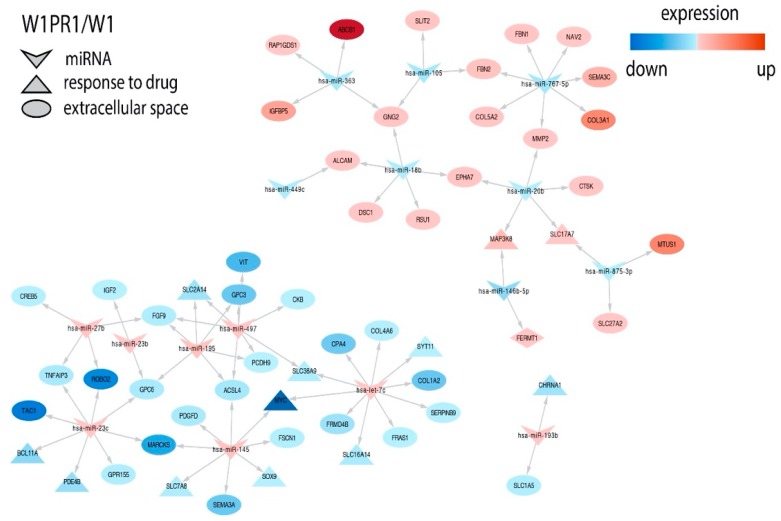
Regulation of selected target genes expression by miRNAs in the W1PR1 cell line.

Many more targets were identified in the PAC and the TOP resistant cell lines and among them were important genes related to drug resistance or other features of cancer cells. In the W1PR1 cell line, we observed inverse correlation between expression of miR-363 and key drug resistant gene *ABCB1* (*ATP binding cassette subfamily B member 1*). Expression of important collagens—*COL3A1* (*collagen type III alpha 1 chain*) and *COL5A2* (*collagen type V alpha 2 chain*)—was correlated with miR-767-5p. Overexpression of receptor tyrosine kinases *EPHA7* (*ephrin type-A receptor 7*) was associated with downregulation of miR-18 and miR-20b. miR-20b and miR-146b-5p downregulation was correlated with upregulation of *MAP3K8* (*mitogen-activated protein kinase kinase kinase 8*). *SEM3A* (*semaphorin 3A*) and *MYC* (*myelocytomatosis viral oncogene homolog*) downregulation was associated with overexpression of miR-145. *MYC* expression was also regulated by let-7c. Upregulation of miR-497 and miR-195 was associated with *PCDH9* (*protocadherin 9*) downregulation (Figure 5).

**Figure 6:**
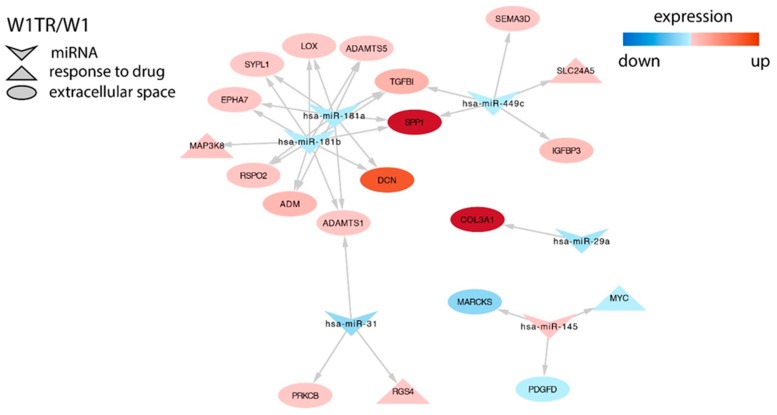
Regulation of selected target genes expression by miRNAs in the W1PR1 cell line.

Changes in different miRNAs were also associated with changes in expression of important drug resistant genes in the W1TR cell line. miR-181a and miR-181b downregulation was associated with increased expression of *SPP1/OPN* (*secreted phosphoprotein 1/osteopontin*), *TGFBI* (*transforming growth factor beta induced*), *LOX* (*lysyl oxidase*), *EPHA7*, *DCN* (*decorin*), and *MAP3K8* (only miR-181b). Expression of *SPP1* was also associated with miR-449c downregulation, and miR-449c downregulation correlated with *SEMA3D* (*semaphorin 3D)* overexpression. Similarly, in the W1PR1 cell line, *MYC* downregulation was associated with overexpression of miR-145. Very high upregulation of *COL3A1* was associated with downregulation of miR-29a in this cell line (Figure 6).

**Figure 7:**
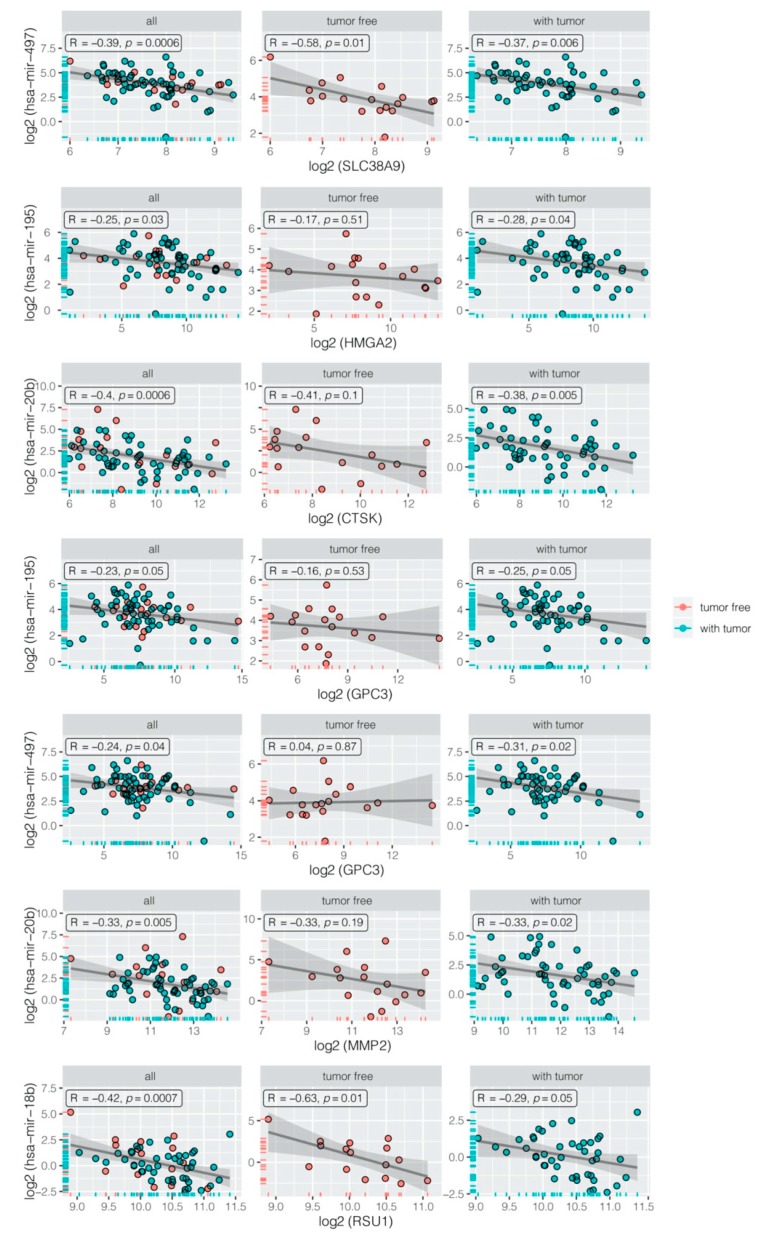
Negative correlation between miRNA and its appropriate target gene in all samples (all), chemotherapy sensitive (tumor free), and chemotherapy resistant patients (with tumor). All of the expression data were log2 transformed to achieve normal distribution.

A negative correlation between miRNAs and their mRNA targets was verified using the data from the The Cancer Genome Atlas (TCGA) database, where we selected mRNA and miRNA expression data for 139 patients treated with cisplatin. It should be noted that this group was not homogeneous because the majority of patients underwent chemotherapy with other cytostatic agents. From the whole group, we received data for 96 patients with person neoplasm cancer status = “with tumor”, considered as chemotherapy resistant patients. A total of 43 patients with person neoplasm cancer status = “tumor free” were considered as chemotherapy sensitive cases. Negative correlations with appropriate R and *p* < 0.05 are presented in Figure 7.

## 3. Discussion

The present study demonstrates the correlation between resistance to cytotoxic drugs and expression of genes encoding microRNAs.

It was reported that changes in miRNAs expression can be related to the development of chemotherapy resistance in solid tumors as well as the development of resistance of cancer cell lines to cytotoxic drugs in vitro [41]. Thus, in the present study, we analyzed changes in the expression of several miRNAs in ovarian cancer cell lines resistant to drugs from the first line (CIS and PAC) as well as from the second line of chemotherapy (DOX and TOP). We were especially interested in changes of miRNAs expression in cancer cells exposed to drugs with different mechanisms of action. In contrast to studies in which only one pair of sensitive/resistant cell lines is tested, in this study, changes in miRNA expression in five drug resistant cell lines with PAC-resistant twin cell lines were examined. It is a unique model for such research.

Among the 27 miRNAs analyzed in this experiment, we could observe significant downregulation of miR-31 and miR-449c in four out of five drug resistant cell lines. Such a significant decrease in expression in cell lines resistant to CIS, TOP, and—especially noteworthy—in both PAC resistant cell lines may indicate a role in the process of resistance to these drugs. This is supported by other studies in which a downregulation of miRNA-31 induced taxane resistance in ovarian cancer cells. miR-31 was downregulated in a PAC resistant cell line, and reintroduction of miR-31 again sensitized them to PAC both in vitro and in vivo [34]. The miR-31 suppressor effect in the ovarian cancer cell line and tissue was confirmed by Hassan M.H. et al. [42]. They observed a high level of miR-31 in the chemosensitive serous ovarian cancer cell line and a decrease in the taxane-resistant line [42]. All these results suggest that downregulation of miR-31 expression may be associated with PAC resistance in ovarian cancer. The potential role of reduced miR-31 expression in CIS resistance can also be assumed. This is demonstrated by the results of our study, in which significant downregulation in the CIS-resistant cell line was noted. This is in line with the results of other studies that showed decreased miR-31 levels in CIS-resistant tumors and the cell line of gallbladder cancer (GBC). In contrast, the ectopic expression of miR-31 in a CIS-resistant cell line causes an increased CIS sensitivity [43]. Because, in our study, for the first time, we observed downregulation of miR-31 expression in a TOP-resistant cell line, the potential contribution of miR-31 with the development of TOP resistance requires further investigation.

Unlike miR-31, expression of miR-449c has not been described in the literature in the context of drug resistance in cancer. Since we demonstrated a downregulation of miR-449c in cell lines resistant to different cytotoxic agents, this may suggest its non-specific role in drug resistance. Previously, we observed ALDH+ stem cell-like population in W1PR1, W1TR, and W1PR2 cell lines [44,45]. Here, we observed downregulation of miR-31 and miR-449c in all these cell lines, and, importantly, the level of these miRNAs downregulation was inversely correlated with the number of ALDH+ cell populations. This suggests the role of miR-31 and miR-449c downregulation in the development of the stem cell population. Lv C. et al. showed that miR-31 plays a critical role in mammary stem cell self-renewal and breast tumorigenesis by regulating the Wnt pathway [46].

Increased expression of miR-1271 in our PAC-, CIS-, and TOP-resistant cell lines contradicts the results of other investigators. Expression of miR-1271 was downregulated in gastric cancer and cell lines in comparison to healthy tissues and cells and was further downregulated in the CIS-resistant cell lines [47]. Glioblastoma patients with a low level of miR-1271 have shorter overall survival than patients with a higher level [48].

We demonstrated significant downregulation of three subsequent miRNAs (miR-105 and miR-767-5p in CIS and PAC-resistant and miR-875-3p in PAC- and TOP- resistant cell lines). Lu G. et al. showed that, in NSCLC (Non-Small Cell Lung Cancer), low miR-105 expression was associated with poor patient survival, suggesting its hypothetical role in chemotherapy resistance [49]. In contrast, in triple negative breast cancer, it was shown that miR-105 was upregulated and correlated with poor patient survival and promoted chemoresistance [50]. However, clinical study does not reflect a simple drug sensitive/resistant cell line model. In contrast, we did not find any data about the role of miR-767-5p and miR-875-3p in drug resistance. Thus, the role of miR-767-5p and miR-875-3p in drug resistance can be considered possible, but drawing specific conclusions requires more thorough research.

Upregulation of two other miRNAs (miR-152 and miR-195) in cell lines resistant to CIS and PAC could indicate their involvement in the regulation of genes associated with resistance to the first line chemotherapy of ovarian cancer. However, in contrast to our study, downregulation of miR-152 and miR-195-5p was observed in CIS-resistant ovarian cancer cell lines [51,52]. On the other hand, the upregulation of miR-195 was observed in the PAC-resistant laryngeal cancer cell line [53] and in docetaxel resistant head and neck squamous cell carcinoma [54]. Thus, the role of these miRNAs in drug resistance could be cell and drug type dependent.

Based on the second research hypothesis assumed in advance, a group of miRNAs whose expression level was changed at least 10-fold in one drug resistant cell line was selected. In this group, as many as nine different miRNAs (miR-10a, miR-146b-5p, miR-203, miR-30a, miR-363, miR-18b, miR-145, miR-195, and miR-99a) showed changes in the expression level in PAC resistant cell lines. However, we could also observe high changes in the expression of single miRNAs in CIS- (miR-195, miR-203), TOP- (miR-29a, miR-145, miR-335), and DOX-resistant (miR-146b-5p, miR-214) cell lines. We are aware that the obtained results cannot clearly indicate the role of these molecules in the development of drug resistance. Some of them were described here for the first time (miR-99a, miR-145, and miR-18b). For others, we searched any literature data describing their involvement in the context of drug resistance or, if not found, at least in the pathogenesis of various cancers.

As the result of literature search, we found that other research teams described the changes in expression levels of the same miRNAs as ours. In contrast to our result, where a decreased level of miR-10a in the PAC-resistant cell line was noted, Sun W. et al. observed an increased expression level in the CIS-resistant A549 lung cancer cell line. Furthermore, miR-10a silencing increases sensitivity of resistance in the cell line to CIS [55]. These differences can result from different drugs and different cancer types used in both studies. Further studies report that low level of miR-146b-5p expression correlates with ovarian cancer progression. In advanced cancer stages (III/IV), the level of miR-146b-5p expression was lower compared to earlier stages (I/II) [56]. In the cell line study, overexpression of miR-146b-5p enhanced ovarian cancer cell sensitivity to PAC and CIS [56]. Thus, miR-146b-5p downregulation seems to play a role in the resistance of ovarian cancer to cytotoxic drugs, especially to PAC.

In opposition to our results (upregulation in PAC and CIS resistant cell lines), Cheng R. et al. showed that, in CIS-resistant NSCLC tumors, miR-203 was downregulated compared with CIS-sensitive tumors [57]. Ectopic expression of miR-203 in the NSCLC cell line increased CIS sensitivity and apoptosis [57]. Overexpression of miR-203 also reversed PAC-resistance in colorectal cancer [58]. Regarding miR-30a and the increase in expression we observed in the PAC-resistant cell line, Sestito et al. showed miR-30a was downregulated in CIS- and PAC-resistant ovarian cancer cell lines [23], and overexpression of miR-30a caused increased sensitivity of ovarian cancer cells to CIS-induced apoptosis [23].

In contrast to our report, Mohamed Z. et al. [59] observed upregulation of miR-363 in the PAC resistance ovarian cancer cell line. Furthermore, overexpression of miR-363 confers PAC resistance, and inhibition of miR-363 restores the response to PAC [59]. In contrast, downregulation of miR-363 was observed in the CIS-resistant hepatocellular carcinoma cell line [60]. A significantly lower expression level of miR-363 was also observed by Cao L. et al. in the CIS-resistant ovarian cancer cell line [61]. Thus, the significance of miR-363 in drug resistance can be cell line dependent.

The next three miRNAs described in this report hypothesized their involvement in resistance to DOX, CIS, and TOP. In the ovarian cancer cell line study, downregulation of miR-29a correlated with increased CIS resistance, while overexpression of miR-29a correlated with sensitized ovarian cancer cell line to CIS [62]. Similar results were obtained by Yang L. et al. in glioblastoma stem cells in which overexpression of miR-29a in CD133+ GBM (glioblastoma multiforme) stem cells effectively reversed the resistance to CIS [63]. Downregulation of miR-29a was also observed in the methotrexate resistant osteosarcoma cell line [64]. Here, we observed downregulation of miR-29a in the TOP-resistant cell line. Thus, it appears that a downregulation of miR-29a could be involved in cytotoxic drug resistance.

In our study, miR-335 was significantly upregulated in the TOP-resistant cell line. In contrast, downregulation of miR-335 was observed in PAC- and CIS- resistant A2780 ovarian cancer cell lines [65]. In the DOX-resistant cell line, we could observe very significant downregulation of miR-214. Downregulation of this miR was also observed in the DOX-resistant urothelial bladder cancer tissues and cells lines [66]. Thus, the miR-214 can be a potential marker of DOX resistance. We, for the first time, observed upregulation of miR-99a and miR-145 and downregulation of miR-18b in the PAC-resistant cell line. Upregulation of miR-99a was observed in CIS-resistant gastric cancer cell lines [67] and vincristine-resistant acute lymphoblastic leukemia (ALL) [68]. In contrast to our study, downregulation of miR-145 was observed in PAC-resistant variants of A2780 and SKOV-3 ovarian cancer cell lines [69]. We did not find any information about the significance of miR-18b in drug resistance in general. Thus, its role in drug resistance requires further investigation.

It is assumed that miRNAs from the same family can play a very similar role in regulation of genes expression. Therefore, as the last step in our research, we analyzed changes in miRNAs expression in the same families. In our study, miR-193a-5p and miR-193b were upregulated in the PAC-resistant cell line, which suggests they can regulate the expression of genes involved in resistance to this drug. As demonstrated by Khordadmehr et al., the miR-193 family (miR-193a-3p, miR-193a-5p, miR-193b3p, and miR-193b-5p) plays an important role in the pathogenesis of ovarian cancer [70]. A similar result was obtained for miR-23 family members, miR-23b and miR-23c. As there are no literature data describing the contribution of these miRNAs as a cluster to cytostatic resistance, at this stage, one can only assume that they may affect the expression of genes associated with PAC resistance. However, the upregulation of miR-23b correlated with better survival of lung cancer patients [71] and, on the other hand, with DOX-resistance in thyroid cancer cells [72]. In another study, downregulation of miR-23b and miR-27b was observed in multidrug resistance protein 1 (MDR) Ehrlich ascites tumor cells [73]. Thus, the role of these miRs in drug resistance requires further investigation. On the other hand, the downregulation of miR-181a and miR-181b in the TOP-resistant ovarian cell line might be an indicator of their involvement in the development of TOP resistance. However, their role in TOP-resistance has not been investigated by others. Decreased expression of miR-181a was observed in the CIS-resistant cervical cancer cell line [74] and miR-181b in the NSCLC cell CIS-resistant cell line [75].

Members of the miR-199 family were described in ovarian cancer. Cheng W. et al. showed that miR-199a-3p and miR-199a-5p overexpression significantly decreased chemoresistance of cancer-initiating cells (CICs) to CIS, PAC, and DOX and reduced mRNA expression of the multidrug resistance gene *ABCG2* in vivo [76]. miR-199b-5p silencing is significantly associated with acquired chemoresistance in ovarian cancer cell lines and cancer tissues [77]. Here, we observed significant downregulation of miR-199a-3p and miR-199b-3p in a DOX-resistant cell line. However, as previously reported, *ABCG2* gene expression did not change in this cell line [38]. Determining the involvement of miR-199 family members in the regulation of drug resistant genes requires further investigation.

As presented above, both our results and studies of other researchers indicate some involvement of specific miRNAs in the process of drug resistance. However, the interpretation of miRNA expression results is much more complicated compared to gene encoding proteins. As an example, the expression of *MDR1* gene encoding P-gp protein is always related to a drug resistant phenotype. In the case of miRNA genes, this phenomenon is more comprehensive. On the one hand, one miRNA can regulate the expression of different mRNAs, and, on the other hand, the same mRNA can be regulated by different miRNAs. This generates a complex network of dependencies.

Considering the high complexity of the process of acquiring resistance by cancer cells and summarizing the results obtained in this study, we believe that the contribution of miR-31, miR-146b, and miR-195 in PAC- and of miR-214, miR-199a-3p, and miR-199a-5p in DOX resistance is highly possible. However, the role of the described miRNAs requires further molecular, cellular, and bioinformatics analysis, which we intend to proceed with in the next step of our study.

To shed more light on the role of investigated miRs in drug resistance, in the second part of our study, we analyzed the correlation between the expression of miRs and their targets. We were mainly interested to see if genes described previously in the context of drug resistance could be regulated by miRNAs, although some other genes were also discussed.

In our previous microarray analysis [38,39,40,78,79], we could observe many more genes with changed expression in PAC- and TOP-resistant cell lines than in CIS- and DOX-resistant ones. This was also reflected by the number of targets to miRNAs identified in the Gene Expression Omnibus (GEO) database in these cell lines. Thus, according to the number of identified targets, we could divide our cell lines into those with a low number of identified targets, represented by CIS- and DOX-resistant cell lines, and those with a high number of identified targets, represented by PAC- and TOP-resistant cell lines.

In the W1CR1 cell line, the only gene that was described in the context of CIS-resistance was *SLC2A14* regulated by miR-195 [38]. The only gene described in the context of drug resistance in the DOX-resistant cell line was *SEMA6A* regulated by miR-875-3p. However, in contrast to our results, downregulation of *SEMA6A* was observed in the DOX-resistant A2780 ovarian cancer cell line [80]. Thus, the role of this miR and its target in drug resistance requires further investigation.

As we described previously [81], the most important gene responsible for PAC resistance in the W1PR1 cell line was *ABCB1* (*MDR1*) encoding the glycoprotein P (P-gp). Here, we observed that upregulation of this gene is related to downregulation of miR-363 in this cell line, which is the first such observation. However, downregulation of miR-363 was associated with *ABCC1* overexpression in oxoplatin hepatocellular carcinoma cell lines [82].

Previously, we reported overexpression of several collagen genes in different drug resistant ovarian cancer cell lines [83]. In this experiment, we could observe that increased expression of *COL3A1* and *COL5A2* inversely correlates with miR-767-5p level. Thus, expression of these two genes seems to be regulated by this miRNA. Similarly, we observed downregulation of miR-767-5p in the W1PR2 cell line accompanied by very strong *COL3A1* upregulation [83]. This further supports the possible role of miR-767-5p in *COL3A1* gene regulation.

Drug resistant cells are characterized by strong signal transduction. Previously, we reported increased total pTYR (phosphotyrosine) level in drug resistant cell lines in comparison to parental cell lines W1 [84]. Here, we observed that upregulation of receptor tyrosine kinase—*EPHA7* and *MAP3K8* kinase—is inversely correlated with miR-18b (*EPHA7*) and miR-18b and miR-20b together (*EPHA7* and *MAP3K8*). Regulation of EPHA7 expression by miR-18b was also described by others [85]. It was reported that signal transduction by the EPHA7 receptor may activate the extracellular-signal-regulated kinase (ERK) signaling pathway that also involves MAP3K8 [86]. Upregulation of EPHA7 was observed in many cancers, including hepatocellular carcinoma, where it was associated with tumor progression, invasiveness, and metastasis [87]. MAP3K8 was described as a mediator of vemurafenib resistance of thyroid cancer stem cells [88].

SEMA3A is a member of the semaphorin family of proteins that play a role in many developmental processes as well as in cancer progression [89]. It is a tumor suppressor gene, and its downregulation correlates with progression of gastric [90] and ovarian cancer [91]. Recently, we described *SEMA3A* downregulation in three PAC-resistant ovarian cancer cell lines [92]. Here we showed that expression of *SEMA3A* can be influenced by miR-145 upregulation in the PAC resistant cell line. Regulation of *SEMA3A* by miR-145 is not surprising because SEMA3A as a direct target of miR-145 was described recently [93]. The upregulation of miR-145 was also discovered to be connected with strong downregulation of the Myc proto-oncogene protein in W1PR1 cell lines. miR-145 was described as a suppressor miRNA that directly targets the *MYC* transcript in esophageal squamous cell carcinoma [94] and ovarian cancer [95]. Moreover, as our analysis shows, the *MYC* downregulation can be due to let-7c upregulation in this cell line as well, which was described previously in prostate cancer cells [96].

Another gene found to be downregulated in the PAC resistant cell line was *PCDH9*, the expression of which correlated with miR-195 and miR-497 upregulation. PCDH9 (protocadherin 9), a member of the protocadherin protein family [97], is a calium-dependent adhesion protein playing a role in neural cell interaction [98]. It is a tumor suppressor gene with decreased expression in ovarian [99] and gastric cancer [100], and its downregulation usually correlates with disease progression and shorter survival. Previously, we observed the *PCDH9* downregulation in three PAC resistant cell lines [92], suggesting its significance in resistance to this cytotoxic agent. However, there are no literature data available describing the regulation of the *PCDH9* gene by miR-195 or miR-497.

The very important role in the TOP-resistant cell line was downregulation of miR-181a and miR-181b, since their downregulation was inversely correlated with upregulation of key genes involved in drug resistance. As in the W1PR1 cell line, in the W1TR cell line, we observed an increased total pTYR level [84]. Additionally, increased *EPHA7* and *MAP3K8* levels were noted. It can be associated with stronger signal transduction, increased pTYR level, and eventual drug resistance. As opposed to the W1PR1 cell line, both genes were regulated here by miR-181a and miR-181b, which is the first such observation described thus far.

We could also observe that both miR-181a and miR-181b regulated *DCN* and *LOX* genes’ expression. DCN (decorin) is a small leucine reach proteoglycan, a part of ECM that binds to collagens and plays an important role in cancer development and metastasis [101]. Regulation of *DCN* by miR-181 was reported in skin and wound healing [102]. LOX (lysyl oxidase) is a secretory protein involved in cross-linking of collagens and elastin in the ECM that results in increased ECM stabilization [103]. Its expression seems to be an important metastatic factor in breast [104] and ovarian [105] cancers, among others. Previously, we described increased expression of LOX in PAC and TOP resistant ovarian cancer cell lines, and its expression was upregulated in ALDH1A1 positive cells [45]. However, the regulation of the *LOX* gene by miR-181a or miR-181b has not been described in literature thus far.

Two other genes important in drug resistance and cancer progression were also regulated by miR from the 181 family as well as miR-449c. TGFBI (transforming growth factor-beta-induced protein) is a collagen I, II, and IV binding protein present in ECM. It has been described as the metastasis promoting protein in ovarian cancer [106], and its expression was correlated with shorter survival of patients with serous ovarian cancer [107]. Recently, we also described TGFBI as a gene related to TOP resistance in three different ovarian cancer cell lines [38]. It was proven that expression of TGFBI is regulated by miR-181a in osteoblasts during differentiation [108].

SPP1 (secreted phosphoprotein 1), also known as osteopontin (OPN), was originally described in bone tissue [109]; however, its expression was also described in different cancers, including ovarian [110], as a protein involved in tumor progression, metastasis, and drug resistance [111]. It was reported that PAC-, DOX-, and CIS-resistance were related to the induction of drug transporters protein expression by SPP1 [112] or blocking caspase [113]. Recently, we also described expression of the SPP1 protein in three TOP resistant cell lines [38]. Similar to our results, the downregulation of miR-181a and the upregulation of *OPN* mRNA were observed in CIS-resistant cervical cancer cells [74], and overexpression of miR-181a led to reduced expression of *OPN* and higher sensitivity to CIS. Regulation of *OPN* by miR-181b was described also in eosinophilia in asthma [114]. In contrast, regulation of *SPP1* by miR-449c has not been described thus far.

As we described previously, the W1TR cell line was characterized by abundant *COL3A1* expression, which was secreted from the cells [83]. *COL3A1* was also described as a protein involved in CIS resistance in ovarian cancer [115]. Here, we observed that upregulation of *COL3A1* inversely correlated with downregulation of miR-29a. In a similar way, upregulation of *COL3A1* was observed in two MTX resistant osteosarcoma cell lines and correlated with miR-29a downregulation. Furthermore, it was proven that miR-29a regulates *COL3A1* expression [64].

## 4. Materials and Methods

### 4.1. Reagents

Cisplatin, Doxorubicyn, Topotekan, and Paclitaxel were obtained from Sigma (St. Louis, MO, USA). RPMI-1640 medium, fetal bovine serum, penicillin, streptomycin, amphotericin B (25 µg/mL), and L-glutamine were also purchased from Sigma. QIazol Lysys Reagent, miRNeasy Mini Kit, and RNeasy MinElute Cleanup Kit were obtained from Qiagen (Hilden, Germany). GeneChip™ miRNA 3.1 Array Strip, FlashTag™ Biotin HSR RNA Labeling Kits, GeneAtlas™ Hybridization, Wash, and Stain Kit for miRNA Arrays were obtained from Affymetrix (Santa Clara, CA, USA).

### 4.2. Cell Lines and Cell Culture

The human primary ovarian cancer cell line W1 was established from the tumor tissue of an untreated 54-year-old Caucasian female patient diagnosed for serous ovarian adenocarcinoma (G3, FIGO IIIc), approval of Bioethical Committee at the Medical University of Poznan of 14 June, 2012, number 699/12. Cells grow as a monolayer, present epithelial morphology, and adherent growth model. Sublines resistant to cisplatin (W1CR), doxorubicin (W1DR), topotecan (W1TR), and paclitaxel (W1PR1 and W1PR2) were obtained by exposure of the W1 cell line to stepwise increasing drug concentrations. Final concentrations of each drug were twofold greater than the concentration in the plasma 2 h after intravenous administration. The cells were 8-, 10-, 20-, 641-, and 967-folds resistant to their selective drugs, respectively, as determined by Cell Proliferation Kit I (MTT) [80]. All cell lines were maintained as a monolayer in complete medium (RPMI-1640 medium supplemented with 10% (*v*/*v*) fetal bovine serum, 2 pMl-glutamine, penicillin (100 U/mL), streptomycin (100 U/mL), and amphotericin B (25 µg/mL)) at 37 °C in a 5% CO2 atmosphere.

### 4.3. miRNA Isolation

miRNA was isolated using a reagents kit from Qiagen according to the manufacturer’s protocol. The RNA was quantified using spectrophotometry by measuring the absorbance values at 260 nm and 280 nm, and the 260/280 nm ratio was used to estimate the level of protein contamination. The 260/280 nm ratios of the samples ranged from 1.8 to 2.0. Four RNA samples from each experimental group were subjected to miRNA expression profiling using microarray method. (*N*/group = 4).

### 4.4. Microarray Preparation, Hybridization, and Scanning

miRNA expression profiling was performed using the microarray approach with Applied BiosystemsTM miRNA 3.1 Array Strip (ThermoFisher Scientific, Waltham, MA, USA). The detailed technical procedure was described earlier [116,117]. Each microarray was designed in accordance with the miRBase Release 17 database, including complementary probes for: 1733 human mature miRNA, 2216 human snoRNA, CDBox RNA, H/ACA Box RNA, and scaRNA, 1658 human pre-miRNA. The full procedure for preparing miRNA for hybridization was performed using the FlashTagTM Biotin HSR RNA Labeling Kit (ThermoFisher Scientific, Waltham, MA, USA). Then, 150 ng of previously isolated miRNA was subjected to the poly(A) tailing and biotin ligation procedure, according to the manufacturer’s protocol. Biotin-labeled miRNA were hybridized to Applied BiosystemsTM miRNA 3.1 Array Strip (20 h, 48 °C). Afterwards, the microarrays were rinsed and stained according to the technical protocol using the Affymetrix GeneAtlas Fluidics Station (Affymetrix, Santa Clara, CA, USA). The array strips were scanned using an Imaging Station of GeneAtlas System (Thermo Fisher Scientific, MA, USA).

### 4.5. Microarray Analysis and miRNA Gene Screening

The initial analysis of the scanned array strips was performed with Affymetrix GeneAtlas Operating Software (Affymetrix, Santa Clara, CA, USA). The quality of the miRNA gene expression data was verified using quality control criteria set by the software. The obtained CEL files from the scanned microarrays were imported for further data analysis using BioConductor libraries from the “R” statistical programming language. The Robust Multiarray Average (RMA) normalization algorithm (implemented in the Affy library) was used to normalize, correct the background, and calculate the expression values of all the tested miRNAs [118]. The biological annotation was obtained from the pd.mirna 3.1 library, where the annotated data frame object was merged with a normalized data set, leading to a complete miRNA data table. Differential expression and statistical evaluation were determined using a linear model for microarray data implemented in the "limma" library [119]. P-values were obtained by the empirical Bayes moderated t-test with false discovery rate (FDR) correction for multiple testing. The algorithm of such a correction was implemented in the R "limma" library. The selection criteria for significantly changed miRNA gene expression were based on the difference between folds greater than absolute five and FDR adjusted *p* value (adj.p.val) < 0.05. The results of such a selection were presented as a scatter plot diagram showing the total number of miRNAs up- and downregulated. Differentially expressed miRNA were also visualized as heat maps and tables. Raw data files were deposited in the Gene Expression Omnibus (GEO) repository at the National Center for Biotechnology Information (http://www.ncbi.nlm.nih.gov/geo/) under the GEO accession number GEO: GSE148251.

### 4.6. miRNA-Target Gene Prediction

SpidermiR package was applied to identify potential target genes for differently expressed miRNA. Differentially expressed miRNAs were used as a query for searching target genes in the following databases: for predicted targets—DIANA, Miranda, PicTar, TargetScan, and for experimentally validated targets—miRTAR, miRwalk [120]. To determine the actual expression value of target genes, mRNA transcriptomic data from our published experiment were used [37,38,39]. Obtained fold change values for mRNA were assigned to the target genes data table. For further analysis, we selected only those target genes for which fold change was inversely correlated with the fold change value of appropriate miRNA (cut-off criteria: fold ±5, adjusted *p* value (adj.p.val.) < 0.05). From the whole set of miRNA-targets pairs, we selected only those that were involved in drug resistance, extracellular matrix, and cancer stem cell biology using the following key words in GO terms: “collagen-containing extracellular matrix”, “extracellular matrix”, “extracellular space”, “response to drug”, and “stem cell”. Interactions between miRNA and target genes in the form of selected GO terms were visualized using Cytoscape 3.7.1 (National Institute of General Medical Sciences, Bethesda, MD, USA) [121].

### 4.7. Analysis of The Cancer Genome Atlas (TCGA) Dataset

The detailed procedure of analysis was described earlier [122]. Briefly, clinical description file, RNAseq, and miRNAseq data (*n* = 307 and 461, respectively) from ovarian serous cystadenocarcinoma patients were downloaded from the public TCGA database [123] using the FireBrowse server (http://gdac.broadinstitute.org/) [124]. Then, the voom algorithm from the “Limma” package was used for data normalization [119]. Using the discriminator "patient.drugs.drug. drug_name = cisplatin", we selected patients treated with cisplatin. Because there was no information on resistance to a given cytostatic in the clinical description file, we used "person_neoplasm_cancer_status" to select patients resistant and sensitive to treatment. We assumed that "person_neoplasm_cancer_status = tumor free" refers to chemotherapy sensitive, whilst "person_neoplasm_cancer_status = with tumor" refers to chemotherapy resistant patients. Negative correlation between appropriate miRNA and its target gene was performed using the Pearson’s product-moment correlation coefficient (PPMCC) test. Paired-log transformed normalized expression values were presented as scatter plots with a line of best fit. The 95% confidence interval level for predictions based on linear models was also shown. Results were presented only for the comparisons with *p* < 0.05

## 5. Conclusions

In summary, we identified a set of potential or already described targets for miRNAs with altered expression in drug resistant cell lines. Some of these targets were previously described as important factors in development of drug resistance and/or cancer progression. The way of regulation of some of these target genes by miRNAs was previously described. Regulation of others requires a detailed study at the molecular level, and significance of this regulation needs to be confirmed in the context of drug resistance.

## Figures and Tables

**Table 1:** List of the miRNA fold changes and false discovery rate (FDR) corrected *p* values (adj.p.val.). Each comparison was performed in relation to W1 cells.

Gene Name	miRBase Accession Number	Fold Change (adj.p.val)				
		W1PR1	W1PR2	W1CR	W1TR	W1DR
hsa-let-7c	MIMAT0000064	5.40 (0.001)	N.S	N.S	N.S	N.S
hsa-miR-100	MIMAT0000098	−5.01 (0.0003)	N.S	N.S	N.S	N.S
hsa-miR-105	MIMAT0000102	−19.22 (4.6 × 10^−7^)	−21.80 (1.5 × 10^−6^)	−15.92 (1.5 × 10^−5^)	N.S	N.S
hsa-miR-10a	MIMAT0000253	−34.42 (2.3 × 10^−7^)	−9.07 (0.0002)	N.S	N.S	N.S
hsa-miR-1271	MIMAT0005796	9.08 (0.004)	N.S	6.24 (0.048)	5.54 (0.049)	N.S
hsa-miR-1285	MIMAT0005876	N.S	5.54 (0.01)	N.S	N.S	N.S
hsa-miR-143	MIMAT0000437	8.81 (0.003)	N.S	N.S	N.S	N.S
hsa-miR-145	MIMAT0000437	12.07 (4.8 × 10^−5^)	N.S	N.S	7.05 (0.002)	N.S
hsa-miR-146b-5p	MIMAT0002809	−39.13 (3.5 × 10^−8^)	N.S	N.S	N.S	−6.93 (0.0003)
hsa-miR-152	MIMAT0000438	18.19 (6.5 × 10^−5^)	16.52 (0.0003)	8.53 (0.0006)	N.S	N.S
hsa-miR-181a	MIMAT0000256	N.S	N.S	N.S	−12.28 (0.0003)	N.S
hsa-miR-181b	MIMAT0000257	N.S	N.S	N.S	−9.39 (1.4 × 10^−7^)	N.S
hsa-miR-18b	MIMAT0001412	−10.22 (0.0006)	N.S	N.S	N.S	N.S
hsa-miR-193a-5p	MIMAT0004614	5.32 (0.01)	N.S	N.S	N.S	N.S
hsa-miR-193b	MIMAT0002819	7.18 (0.01)	N.S	N.S	N.S	N.S
hsa-miR-195	MIMAT0000461	13.94 (0.003)	N.S	6.67 (0.046)	N.S	N.S
hsa-miR-199a-3p	MIMAT0000462	N.S	N.S	N.S	N.S	−23.09 (0.01)
hsa-miR-199b-3p	MIMAT0000464	N.S	N.S	N.S	N.S	−28.18 (0.02)
hsa-miR-203	MIMAT0000264	14.45 (6.9 × 10^−6^)	N.S	22.33 (1.5 × 10^−5^)	N.S	N.S
hsa-miR-205	MIMAT0000465	N.S	N.S	N.S	N.S	6.23 (0.004)
hsa-miR-20b	MIMAT0000466	−8.87 (6 × 10^−8^)	N.S	N.S	N.S	N.S
hsa-miR-214	MIMAT0000271	N.S	N.S	N.S	N.S	−26.05 (0.001)
hsa-miR-23b	MIMAT0000468	6.06 (0.0009)	N.S	N.S	N.S	N.S
hsa-miR-23c	MIMAT0000469	7.47 (0.02)	N.S	N.S	N.S	N.S
hsa-miR-27b	MIMAT0000470	9.21 (0.0003)	N.S	N.S	N.S	N.S
hsa-miR-29a	MIMAT0000086	N.S	N.S	N.S	−22.04 (0.0009)	N.S
hsa-miR-30a	MIMAT0000087	N.S	14.82 (0.001)	N.S	N.S	N.S
hsa-miR-31	MIMAT0000089	−217.59 (4.6 × 10^−5^)	−51.72 (0.003)	−13.51 (0.048)	−36.90 (0.008)	N.S
hsa-miR-335	MIMAT0000765	N.S	N.S	N.S	21.31 (0.0009)	N.S
hsa-miR-363	MIMAT0000707	−16.37 (1.55 × 10^−7^)	N.S	N.S	N.S	N.S
hsa-miR-4269	MIMAT0016897	N.S	N.S	N.S	N.S	−5.13 (0.04)
hsa-miR-449c	MIMAT0016897	−11.97 (0.006)	−8.09 (0.04)	−5.61 (0.049)	−6.53 (0.047)	N.S
hsa-miR-4721	MIMAT0019835	N.S	7.18 (0.03)	N.S	N.S	N.S
hsa-miR-497	MIMAT0002820	8.22 (0.02)	N.S	N.S	N.S	N.S
hsa-miR-501-5p	MIMAT0002872	N.S	−5.60 (0.002)	N.S	N.S	N.S
hsa-miR-572	MIMAT0003237	N.S	6.23 (0.04)	N.S	N.S	N.S
hsa-miR-708	MIMAT0004926	N.S	N.S	5.52 (0.007)	N.S	N.S
hsa-miR-767-5p	MIMAT0003882	−20.61 (9 × 10^−6^)	−19.99 (3 × 10^−5^)	−13.95 (0.0004)	N.S	N.S
hsa-miR-875-3p	MIMAT0004923	−7.93 (0.02)	−7.32 (0.048)	N.S	N.S	−6.28 (0.049)
hsa-miR-99a	MIMAT0000097	11.02 (0.004)	N.S	N.S	N.S	N.S

## Abbreviations

CIS	Cisplatin
PAC	Paclitaxel
DOX	Doxorubicin
TOP	Topotecan
FIGO	fr. *Fédération internationale de gynécologie et d’obstétrique*
ABCB1 (MDR1)	ATP-binding cassette subfamily B member 1 (multidrug resistance protein 1)
W1PR1, W1PR2, W1CR, W1TR, W1DR	Resistant Variants of W1 Sensitive Ovarian Cancer Cell Line
ECM	Extracellular Matrix
FDR	False Discovery Rate
GEO	Gene Expression Omnibus
GO	Gene Ontology
COL3A1	Collagen Type III Alpha 1 Chain
COL5A2	Collagen Type V Alpha 2 Chain
MYC	Myelocytomatosis Viral Oncogene Homolog
EPHA7	Ephrin Type-A Receptor 7
MAP3K8	Mitogen-Activated Protein Kinase Kinase Kinase 8
LOX	Lysyl Oxidase
EPHA7	Ephrin type-A receptor 7
DCN	Decorin
MAP3K8	Mitogen-Activated Protein Kinase 8
SEM3A	Semaphorin 3A
PCDH9	Protocadherin 9
SPP1/OPN	Secreted Phosphoprotein 1/Osteopontin
TGFBI	Transforming Growth Factor Beta Induced
